# Constrictive Pericarditis and Protein-Losing Enteropathies: Exploring the Heart–Gut Axis

**DOI:** 10.3390/jcm13175150

**Published:** 2024-08-30

**Authors:** Lucia Ilaria Birtolo, Endrit Shahini

**Affiliations:** 1 Cardiology Unit, National Institute of Gastroenterology–IRCCS “Saverio de Bellis”, 70013 Castellana Grotte, Italy; ilaria.birtolo@irccsdebellis.it; 2 Department of Clinical, Internal, Anesthesiology and Cardiovascular Sciences, Umberto I Hospital, Sapienza University of Rome, 00185 Rome, Italy; 3 Gastroenterology Unit, National Institute of Gastroenterology–IRCCS “Saverio de Bellis”, 70013 Castellana Grotte, Italy

**Keywords:** enteropathy, non-coeliac disease, protein loss, hypoalbuminemia, hypoproteinemia

## Abstract

**Background/Objectives**: Constrictive pericarditis very rarely causes protein-losing enteropathy (PLE) induced by secondary intestinal lymphangiectasia. This study thoroughly reviewed the literature to shed light on the clinical management of PLE provoked by intestinal lymphangiectasia following constrictive pericarditis. **Methods**: We performed a PubMed search using the keywords enteropathy, protein-losing enteropathy, pericarditis, acute pericarditis, pericardial effusion, recurrent pericarditis, constrictive pericarditis, noninfectious pericarditis, idiopathic pericarditis, and infective pericarditis, with only English-language publications included. **Results**: Although constrictive pericarditis is primarily idiopathic, less common causes include infectious etiologies, connective/autoimmune tissue disorders, previous cardiac surgery, congenital syndromes, and cancer. On the one hand, PLE secondary to intestinal lymphangiectasia may cause a severe cellular immune deficiency that could raise infection hazards due to lymphocytopenia and hypogammaglobulinemia. On the other hand, lymphocytopenia may cause anergy and mask an underlying tuberculous etiology of constrictive pericarditis. Cardiac catheterization is the most useful diagnostic tool for constrictive pericarditis, though it may be misdiagnosed in rare cases. The videocapsule endoscopy and double-balloon enteroscopy techniques can detect small bowel lymphangiectasias distal to the Treitz ligament. MRI or a CT scan helps confirm constrictive pericarditis, visualize lymphangiectasias, and reveal features specific to the underlying etiology of PLE. Radioisotopic techniques may ensure PLE diagnosis in challenging cases, whereas fecal alpha1-antitrypsin can estimate gastrointestinal protein loss. **Conclusions**: Constrictive pericarditis is rarely associated with PLE. The cardio-intestinal abnormalities of PLE caused by constrictive pericarditis are frequently reversed following a complete pericardiectomy, though its ability to invert severe hypoalbuminemia is currently unknown.

## 1. Introduction

Constrictive pericarditis is generated by chronic scarring and loss of pericardial elasticity, with unexplained reasons in 33% to 42% of cases, resulting in impaired diastolic filling [1,2]. The etiology of constrictive pericarditis remains unknown in many cases, although previous cardiac surgery or radiation therapy is one of the major causes [3].

Intestinal lymphangiectasia is classified as a primary disorder of the gastrointestinal (GI) tract if the malformations are intrinsic to the peripheral lymphatic system and secondary if the lymphatic flow is damaged by external lymph vessel obstacles or cardiovascular disease [4]. Notably, intestinal lymphangiectasia is associated with protein-losing enteropathy (PLE), which can be diagnosed by ruling out other causes of hypoproteinemia, such as diarrhea and malabsorption.

Constrictive pericarditis is rarely responsible for PLE due to a secondary form of intestinal lymphangiectasia, being characterized by abnormal and dilated intestinal lymphatic channels in the mucosa, submucosa, and serosa, as well as excessive serum protein loss into the GI tract [2,4,5,6]. Mild steatorrhea can occur, and its provenience could be due to impaired fat absorption caused by delayed intestinal lymphatic transport or increased endogenous fecal fat [7].

The pathogenic mechanisms that originate PLE in people with constrictive pericarditis or other cardiac diseases are not fully understood. High systemic venous pressure seems to be a prerequisite, as it is associated with increased lymphatic flow and hydrostatic pressure in the thoracic duct [5,8].

Generally, the PLE rate of serum protein loss into the GI tract exceeds the body’s synthesis capacity, resulting in secondary hypoproteinemia, which manifests mainly as variable peripheral edema, with or without ascites and GI symptoms [7,9,10]. Malabsorption and malnutrition in children can be severe, resulting in slowed growth and development [4].

Previous research has also found an association of immunologic deficiency with PLE, including hypogammaglobulinemia, lymphocytopenia, cutaneous anergy, decreased in vitro lymphocyte proliferative responses to a variety of stimuli, and an increased risk of neoplasia [11,12].

To the best of our knowledge, this is the first holistic review examining the clinical management of PLE provoked by intestinal lymphangiectasia following constrictive pericarditis, using a detailed analysis of all published pediatric and adult cases.

## 2. Materials and Methods

We searched the PubMed database for English articles that described the potential hazards and outcomes of PLE in patients with constrictive pericarditis. We used the following MESH terms: ((((((((((enteropathy) OR (protein-losing enteropathy)) AND (pericarditis)) OR (acute pericarditis)) OR (pericardial effusion)) OR (recurrent pericarditis)) OR (constrictive pericarditis)) OR (noninfectious pericarditis)) OR (idiopathic pericarditis)) OR (infective pericarditis). We found 27,351 articles and finally chose twenty-nine papers (from 1960 to 2022) after excluding those unrelated.

## 3. Results

### 3.1. Pediatric Cases

Table 1 summarizes the most significant clinical characteristics of pediatric patients affected by PLE syndrome due to constrictive pericarditis.

Jiménez-Díaz et al. first described PLE in 1960, citing the case of a young boy with hypoalbuminemia, steatorrhea, and hypocalcemia [13]. Briefly, after a pericardiectomy, serum albumin increased and fecal fat decreased. Then, in 1961, Davidson et al. [14] independently reported three cases of young patients with constrictive pericarditis and hypoalbuminemia, using radioactive 131I-polyvinylpyrrolidone to prove a PLE diagnosis. All of the latter patients’ hypoalbuminemia and abnormal intestinal albumin loss resolved after surgical treatment, and venous pressure returned to normal, as did the radioisotopic test. Subsequently, Plauth, W.H. et al. [15] reviewed a series of constrictive pericarditis in 1964. They found that in seven [7] of the eight children, a pericardiectomy resulted in a complete response to the excessive GI protein loss. Nevertheless, despite the absence of clinical evidence of recurrent constrictive pericarditis, abnormal protein loss persisted in eight patients for several years following a successful pericardiectomy [15].

In a 14-year-old male (Kumpe, D.A. et al., 1975), radiologic and clinical abnormalities were reversed after a pericardiectomy [9]. He developed acute abdominal pain, diarrhea, and lower extremity edema, with severe hypoalbuminemia. Intravenous 51Cr albumin demonstrated increased protein loss into the gut, while preoperative small bowel series and an exploratory laparotomy with liver and small bowel biopsies revealed intestinal lymphangiectasia. Cardiac catheterization displayed typical findings of constrictive pericarditis of unknown etiology, as confirmed after the pericardiectomy [9]. Peripheral edema and albumin turnover returned to normal within six months of the pericardiectomy, as did his cardiac function and intestinal histology [9].

Notably, among five other similar cases represented by the same author, anergy was present in one of them. The postulated mechanism of underlying anergy was lymphocyte leakage into the GI lumen via dilated lymphatics [9]. In such cases, a negative Mantoux test does not rule out the tuberculous causality of the pericarditis. Nonetheless, this condition returned to normal after a pericardiectomy [9]. Correcting the PLE resulted in substantial clinical improvement in the four patients who underwent a pericardiectomy [9].

Additionally, the established reversal of immunologic deficiency in intestinal lymphangiectasia may reinforce the assumption that the immune defect in PLE may be due to excessive lymphocyte and immunoglobulin loss into the GI tract. In this regard, Nelson, D.L. et al. (1975) reported that a 15-year-old male experienced acute abdominal pain, diarrhea, scrotal swelling, edema, and dyspnea [16]. Small bowel and lymph node biopsies revealed dilated submucosal lymphatic vessels and paracortical lymphocytic depletion. The increased 51Cr albumin stool clearance led to a diagnosis of secondary intestinal lymphangiectasia with PLE, and the patient underwent a specific diet therapy. Then, thoracentesis produced chylous fluid, whereas the superior vena cava cinegram revealed substantial thickening of the right heart and severely compromised diastolic expansion of the right ventricular [16]. The patient was eventually diagnosed with constrictive pericarditis, secondary intestinal lymphangiectasia, and PLE, as well as immunological deficiencies, cutaneous anergy, impaired allograft rejection, and decreased lymphocyte proliferative responses [16]. Following a pericardiectomy, intestinal lymphangiectasia and PLE resolved, whereas immune function gradually improved [16].

Occasionally, the serological presence of autoantibodies reactive with human intestinal epithelial cells may be associated with total jejunal villous atrophy [17]. An underlying non-coeliac autoimmune disease can explain the intestinal damage in such cases that are unresponsive to gluten withdrawal and may necessitate a differential diagnosis. In 1984, Savilahti, E. et al. depicted the fatal course of a diabetic 5-year-old girl who presented with persistent diarrhea and weight loss and showed symptoms of arthritis and recurrent pericarditis on cardiac exams [17]. She had high antinuclear antibody titers, but her antigliadin antibody titer revealed the absence of IgA antibodies and HLA B8 and increased levels of IgG [17]. A jejunal biopsy specimen displayed total villous atrophy and low mitotic activity but regular crypt lengths. A low titer of antibodies (1:5) against human mature intestinal epithelial cells was discovered [17]. Unfortunately, the young patient did not respond to a gluten-free diet, total parental nutrition, or prednisone/cyclophosphamide, and she died of mycotic sepsis, as confirmed by autopsy with no proof of ulcerative jejunitis [17]. The jejunal lesion was not crypt hyperplastic villous atrophy, as seen in childhood coeliac disease. However, the findings resembled those seen in the jejunal mucosa of adult patients with unresponsive coeliac disease and one fatal case with anti-intestinal antibodies [17]. As a result, jejunal villous atrophy was not driven by raised cellular damage but rather by the inhibition of crypt cell mitosis. Nevertheless, this case appears to differ from the others because the patient does not appear to be affected solely by constrictive pericarditis.

Similarly, jejunal villous atrophy has been previously reported in 6 cases among 71 adult patients with various autoimmune diseases [18]. Only two cases responded to a gluten-free diet, while the jejunal atrophy was considered a part of the autoimmune disease in the others [18].

In 2016, Peters B. et al. reported on a 10-year-old female with consanguineous parents who had congenital camptodactyly, swollen and painful joints, and recurrent respiratory infections [19]. Laboratory tests indicated persistent hypoproteinemia and secondary immunodeficiency caused by hypogammaglobulinemia. Increased stool alpha-1 antitrypsin clearance supported PLE. She also had hepatomegaly with portal hypertension and aspecific fibrosis on histology secondary to chronic venous congestion [19]. Echocardiography suggested constrictive pericarditis, but cardiac catheterization did not confirm it. Ultrasound and X-ray examinations of the joints combined with a puncture of the synovial fluid results, together with clinical symptoms and the consanguinity, suggested camptodactyly-arthropathy-coxa vara-pericarditis (CACP) syndrome [19]. CACP syndrome is a rare autosomal recessive disease with high clinical variability. CACP syndrome lacks glycoprotein lubricin, produced via the proteoglycan-4 gene (PRG4) transcription [19]. Lubricin is a glycoprotein expressed in distinct tissues such as joints, pericardial and pleural cavities, liver, kidneys, and skeletal muscles. Noninflammatory pericarditis can occur in up to 30% of cases [19]. A pericardiectomy produced positive results, as evidenced by the normalization of echocardiographic measurements [19]. Finally, genetic testing identified a pathogenic mutation within the repeat sequence in exon 7 of the PRG4 gene.

**Table 1:** Clinical characteristics pediatric patients affected by protein-losing enteropathy (PLE) conditions secondary to constrictive pericarditis.

AUTHOR/YEAR	Kumpe, D.A., 1975 (Ref. [9])	Nelson, D.L., 1975 (Ref. [16])	Savilahti, E., 1985 (Ref. [17])	Peters, B., 2016 (Ref. [19])	Schmitt, E.G., 2021 (Ref. [3])	Xi, Y., 2022 (Ref. [20])
**AGE (years)/GENDER**	14-M	15-M	5-F	10-F	14-F	14-M
**COMORBIDITIES**	N	cutaneous anergy, immunologic deficiency	diabetes, autoimmune jejunitis, unexplained immunodeficiency	congenital camptodactyly: coxa vara, osteopenia and flattened joints (X-ray)	N	pulmonary tuberculosis
**ONSET OF SYMPTOMS**	1 month	1 year	3 months	NA	3 weeks	NA
**ENDOSCOPY**	Y	Y	Y	NA	NA	NA
**VIDEOCAPSULE**	N	N	N	N	N	N
**PLE LOCATION**	Jejunum and ileum	Jejunum	Jejunum	NA	NA	NA
**INTESTINAL HISTOLOGY**	intestinal lymphangiectasia	dilated submucosal lymphatic vessels and paracortical lymphocytic depletion (lymph node biopsies)	total villous atrophy, reduced crypt cell proliferation	NA	NA	NA
**LIVER HISTOLOGY**	no cirrhosis	N	N	liver fibrosis	N	liver fibrosis
**X-RAY—CHEST**	pulmonary venous distention, interstitial pulmonary oedema, pleural effusions	N	N	N	mediastinum mass	N
**X-RAY—BARIUM ENEMA**	diffusely thickened mucosal folds	upper GI: mucosal edema	N	N	N	N
**STOOL ALPHA1-ANTITRYPSIN CLEARANCE**	N	N	N	increased	increased	N
**RADIOISOTOPIC TECHNIQUES**	51Cr albumin stool clearance, increased	51Cr albumin stool clearance, increased	N	N	N	Tc-GSA scintigraphy, radionuclide accumulation in the intestine
**ECG**	NA	low voltage in all leads	typical T-wave changes	NA	NA	NA
**ECOCARDIOGRAPHY**	N	N	pericardial effusion	pericardialeffusion	thickened pericardium	decreased cardiac function
**CT SCAN**	N	N	N	N	thickened pericardium	lung consolidation (enlarged mediastinal lymph nodes), ascites, pleural/pericardial effusions
**MRI**	N	N	N	thickened pericardium	thickened pericardium	N
**RIGHT HEART CATHETERIZATION**	Y	Y	N	Y	Y	N
**PERICARDIECTOMY**	Y	Y	N	Y	N	Y
**CONSTRICTIVE PERICARDITIS—HISTOLOGY**	Y	Y	N	Y	N	Y
**ETIOLOGY**	unknown	unknown	underlying autoimmune disease	CACP syndrome	inflammatory myofibroblastic tumor (mediastinum)	tuberculosis
**RESPONSE TO PERICARDIECTOMY**	edema disappeared, radioactive albumin turnover returned to normal, as did his cardiac catheterization data and intestinal biopsy after 6 months	intestinal lymphangiectasia and PLE reversed, immune function gradually improved after 6 months	no surgery: unresponsive to gluten-free diet, total parental nutrition, immunosuppression, death of mycotic sepsis	peripheral edema and joint swellings disappeared in one year	no surgery: despite a temporary positive clinical response to medical treatment, she developed clinical signs of right heart failure on follow-up	asymptomatic, serum albumin levels increased during the subsequent 2 years

N: No; Y: yes; NA: not available; PLE: protein-losing enteropathy; GI: gastrointestinal; Tc-GSA: technetium-99m diethylenetriamine pentaacetic acid galactosyl human serum albumin; ECG: electrocardiogram; MRI: magnetic resonance imaging; CACP: camptodactyly-arthropathy-coxa vara-pericarditis syndrome.

Subsequently, Schmitt, E.G. et al. depicted a 14-year-old girl who presented with a recent history of progressive lower extremity edema, abdominal distension, weight gain, hypogammaglobulinemia, and severe lymphocytopenia [3]. Finally, based on distinct cardiac exams and an elevated stool alpha-1-antitrypsin level, the young patient was diagnosed with PLE secondary to constrictive pericarditis caused by an inflammatory myofibroblastic tumor of the mediastinum as confirmed by surgical pathology [3]. After tumor mutation analysis, she was eventually treated with an mTOR inhibitor (everolimus), which indicated a phosphatase and tensin homolog (PTEN) mutation. However, due to the tumor’s location and extent, the patient developed clinical signs of right heart failure on follow-up, prompting pericardiectomy [3]. 

Xi, Y. et al. (2022) reported a 5-year-old boy with a strongly positive tuberculin skin test who developed lower extremity edema, serum hypoalbuminemia, and proteinuria [20]. Following a computerized tomography (CT) diagnosis of pulmonary tuberculosis, the patient started an 8-month cycle of isoniazid, rifampin, and pyrazinamide. However, his serum albumin level did not normalize [20]. Ten years later, the young patient displayed acute diarrhea, persistent hepatosplenomegaly with hypoalbuminemia, and recurrent/severe hematuria [20]. Echocardiography and technetium-99m-diethylenetriaminepentaacetic acid-galactosyl-human serum albumin (Tc-GSA) scintigraphy showed reduced cardiac function and determined the intestine as the site of protein leakage in PLE. A liver biopsy indicated the presence of fibrosis [20]. The patient was diagnosed with PLE due to tuberculosis-related constrictive pericarditis. After pericardiectomy, the patient was asymptomatic and returned to normal albumin levels [20]. 

Ultimately, in 2022, Shah NC et al. reported a case of a 2-year-old child who developed PLE as a result of constrictive pericarditis [21]. Cardiac MRI revealed pericardial thickening, and subsequent pericardiectomy enhanced clinical outcomes and resolved PLE [21].

### 3.2. Adult Cases

Table 2 summarizes the most significant clinical characteristics of the adult patients affected by PLE syndrome due to constrictive pericarditis.

In 1963, Petersen VP et al. described a 25-year-old man with constrictive pericarditis with secondary PLE who did not respond sufficiently to pericardiectomy [7]. This patient complained of chronic hepatomegaly, dyspnea, ascites, and edema, whereas cardiac exams confirmed a constrictive pericarditis. After pericardiectomy, he showed excellent clinical improvement [7]. Regardless, postoperative heart catheterization and GI radiology displayed only a partial response. The radioisotopic lower albumin turnover indicated a limited capacity for albumin synthesis due to reduced liver function secondary to cardiac dysfunction [7]. Likewise, studies on thoracic duct lymph using oral ingestion of oleic acid labeled with 131I revealed a significantly increased production of lymph of small bowel origin with a low protein content. Unfortunately, the patient died during his last hospitalization due to persistent vomiting and severe dyspnea, and an autopsy revealed extensive fibrous adhesions in the mediastinum and pleural cavities, as well as calcified fibrous tissue surrounding the heart [7]. Similarly, intestinal histology indicated that lymphangiectasias were mainly localized in the jejunum.

In 1965, Wilkinson P et al. described a 59-year-old black male who had a history of congestive heart failure caused by arteriosclerosis and presented with progressive dyspnea, hypoalbuminemia, and anasarca [22]. Radioisotopic techniques using 131I-albumin and peroral jejunal biopsy displayed excessive protein loss into the GI tract and intestinal lymphangiectasias. Cardiac catheterization showed chronic constrictive pericarditis and raised mean pressures in all cardiac chambers [22]. Following pericardiectomy, which revealed a histologic picture negative for any cause of pericarditis, the patient’s congestive heart failure gradually improved, the PLE disappeared, and the bowel histology returned to normal, allowing him to discontinue therapy [22]. However, liver function did not fully recover on the long-term follow-up, and mild hypoalbuminemia developed in the absence of cirrhosis [22].

In 1991, Müller, C. et al. reported a case of PLE where constrictive pericarditis was diagnosed solely through cardiac magnetic resonance (CMR) and missed by right heart catheterization [5]. In detail, a 41-year-old man presented with peripheral edema caused by hypoalbuminemia. Increased fecal excretion of 51-chromium-labeled albumin and alpha1-antitrypsin clearance indicated protein loss via the GI tract [5]. Endoscopic biopsies from the deep jejunum were histologically normal. PLE was suspected to be caused by constrictive pericarditis, but right heart catheterization did not confirm this. CMR revealed moderate thickening of the pericardium and tubular-shaped right ventricle, indicating constrictive pericarditis [5]. After pericardiectomy, edema resolved, serum protein levels normalized, and the right atrium and inferior vena cava reduced in diameter. One year after surgery, there was still a little enteric protein loss, likely due to a partial pericardiectomy that did not fully address the cardiac filling impediment and PLE [5].

Nikolaidis, N. et al. (2005) presented the case of a 76-year-old man with an atypical clinical presentation of constrictive pericarditis of doubtful infectious origin, with PLE as the primary manifestation [1]. He underwent a right nephrectomy five decades before due to tuberculous nephritis. The patient had a history of atrial fibrillation and congestive heart failure, as well as diabetes mellitus, dating back nearly two decades. He complained about chronic peripheral edema and mild dyspnea [1]. The presence of hypoproteinemia in the absence of kidney or liver disease, as well as malnutrition, suggested PLE. Also, a duodenal histopathologic examination demonstrated markedly dilated lymphatics compatible with intestinal lymphangiectasia; Tc-GSA scintigraphy proved the loss of the radionuclide inside the intestinal lumen. This patient was successfully treated via a pericardiectomy [1]. Additionally, given the prior history of tuberculous nephritis, a pericardial biopsy, the most sensitive test for excluding tuberculosis pericarditis, showed no such evidence [1]. 

Meijers BK et al. published a case report in 2006 of a 74-year-old male who presented with progressive dyspnea and weight gain and was later diagnosed with constrictive pericarditis following coronary artery bypass grafting complicated by right-sided heart failure [23]. PLE aggravated edema formation, leading to hypoalbuminemia. Ultrasound and simultaneous pressure recording of both ventricles suggested a diagnosis of constrictive pericarditis. Tc-GSA scintigraphy allowed for the imaging of intestinal protein loss [23]. Following surgery, the PLE condition, the cardiac illness, and serum albumin levels significantly improved.

A videocapsule endoscopy helps detect intestinal lymphangiectasia by examining the entire small bowel and determining its location. In this regard, in 2006, Chamouard P et al. presented two cases with a diagnosis of PLE accomplished using a videocapsule endoscopy [8]. The second case concerned a 35-year-old woman with PLE and lymphatic abnormalities induced by idiopathic chronic constrictive pericarditis. She had a history of pleuropericarditis and was hospitalized with recurrent leg edema and pleural effusions, as well as hypoalbuminemia, lymphocytopenia, and low immunoglobulin levels [8]. A videocapsule endoscopy revealed mucosal oedema in the jejunum and white curved lines. CMR revealed a thickened pericardium and cardiac catheterization proved to be the diagnosis of constrictive pericarditis [8]. After subtotal pericardiectomy, the histological examination revealed intense and partially non-inflammatory calcified fibrosis, and two months later, serum albumin levels were normal.

Kikuchi S et al. (2013) described the case of a man in his late seventies hospitalized for abnormal liver function tests, right-sided pleural effusion, and worsening leg oedema [2]. After excluding other secondary etiologies, the low albumin levels were secondary to PLE. The Tc-GSA scintigraphy confirmed the suspect since a radionuclide accumulation in the intestines was documented. Endoscopic and histological examinations ruled out GI amyloidosis and intestinal lymphoma, but cardiac CT and two-dimensional echocardiography revealed thickened pericardium, indicating chronic pericarditis [2]. Pericardiectomy restored albumin to normal levels and alleviated all clinical symptoms.

Finally, in 2016, Moriyama H et al. documented a case of a 38-year-old male who had previously undergone mitral valve replacement for severe mitral regurgitation and infective endocarditis in the previous decade and presented with progressive diarrhea and oedema of lower extremities [6]. In addition to hypoalbuminemia, Tc-GSA scintigraphy revealed intestinal radionuclide accumulation. A complete endoscopic look of the GI tract and lymphangiography excluded abnormalities. A CT scan showed bilateral pleural effusion, ascites, and thickened pericardium. Also, transthoracic echocardiography displayed a thickened and calcified pericardium, atrial dimensional enlargement, and mild mitral valve regurgitation [6]. Following that, right heart catheterization confirmed the diagnosis of PLE secondary to constrictive pericarditis. The pericardiectomy was successful, and the patient’s recovery was uneventful.

## 4. Comparisons between Adult and Pediatric Cases

When comparing pediatric and adult cases, some differences must be considered. To begin with, the etiology of constrictive pericarditis was better defined in pediatrics (i.e., autoimmune, CACP syndrome, myofibroblastic tumor, or tuberculosis) than in adults, where the etiology was often unknown.

In most cases, both populations experienced similar clinical symptoms of peripheral oedema and weight gain. Pediatric patients, on the other hand, had a faster onset of symptoms and were more likely to experience diarrhea than adults, who experienced fatigue and respiratory symptoms relatively more frequently. Interestingly, the prevalence of hypoalbuminemia was comparable between the two groups, whereas lymphocytopenia and hypogammaglobulinemia were more common in pediatric cases.

Both populations had similar intestinal pathological changes, primarily lymphatic vessel dilatation in the villi of jejunum.

Additionally, in terms of the prognostic impact of pericardiectomy, in pediatric cases, there was a sharp reversal of clinical signs and symptoms, as well as intestinal lymphangiectasia and PLE conditions during surveillance ranging from 6 months to 2 years. Among the pediatric cases who did not undergo pericardiectomy, one patient died of mycotic sepsis, and the other one developed clinical signs of right heart failure on long-term surveillance.

For adults, the same positive clinical response was confirmed in the short-term follow-up following a complete pericardiectomy, although in a young adult concomitantly affected by a cardiac cirrhosis, a cardiac inflow-stasis persisted and developed after six years and then he died of cardio-intestinal complications [7]. The onset of cardiac cirrhosis may have complicated the clinical course of the cardio-intestinal disease, particularly in a historical context (1963) when diagnostic and therapeutic tools for the management of decompensated liver cirrhosis were far more limited than they are nowadays.

Furthermore, in another young adult case, there was a small enteric protein loss one year after surgery, most likely due to the patient’s subtotal pericardiectomy, which may have resulted in incomplete healing of the constrictive pericarditis [5].

## 5. Discussion

This review aimed to comprehensively examine the clinical management of protein-losing enteropathy provoked by intestinal lymphangiectasia following constrictive pericarditis, using a detailed analysis of all published pediatric and adult case cases. The present review shows that (i) constrictive pericarditis is rarely associated with PLE, and (ii) the cardio-intestinal abnormalities of PLE caused by constrictive pericarditis are frequently reversed following a complete pericardiectomy. Previous research on the cardio-intestinal axis has primarily focused on the link between intestinal flora and cardiovascular disease. Constrictive pericarditis, a chronic inflammatory condition marked by pericardial calcification and fibrinoid exudation, raises serious concerns about its etiology. Alongside abnormal lymphatic system reflux and elevated venous pressure, it is critical to investigate the potential role of immune regulation and immune-related factors in the occurrence of protein-losing enteropathy.

Primary intestinal lymphangiectasia is a congenital lymphatic system disorder characterized by dilated lymphatic vessels in the intestinal villi and the mesentery serosal [4,8]. This condition is associated with PLE syndrome, in which the intestinal epithelium becomes compromised and interstitial fluid leaks into the gut lumen [4]. Therefore, PLE results in an uncompensated loss of plasma proteins in the intestine with reduced albumin, hypogammaglobulinemia, lymphocytopenia, nutritional deficiencies, infections, and GI symptoms such as diarrhea, steatorrhea, abdominal pain, and vomiting [4].

**Figure 1:**
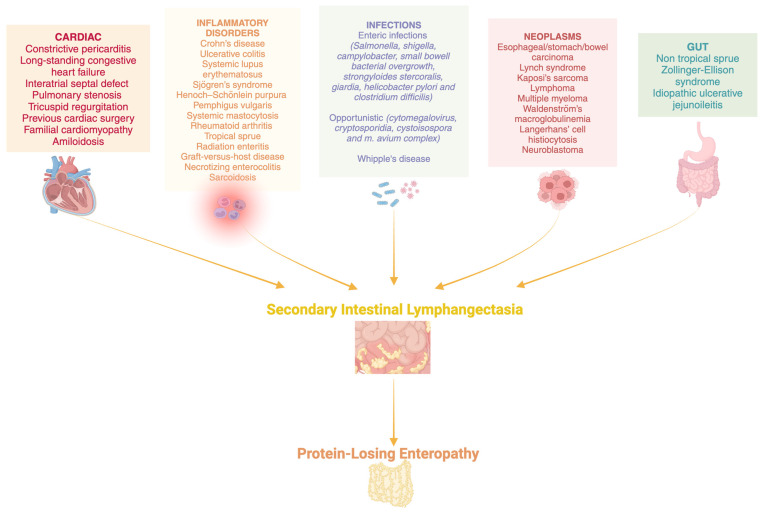
Most prevalent gastrointestinal and not etiologies of secondary intestinal lymphangiectasias associated with protein-losing enteropathy (PLE).

At the same time, cirrhosis and nephrosis, which can likewise induce hypoproteinemia, should be excluded, along with several other etiologies such as cardiac, inflammatory, infectious, and other GI disorders that could provoke secondary forms of intestinal lymphangiectasia (Figure 1) [2,4,8,9].

Distinct cardiovascular diseases that cause high venous pressure over time and may trigger secondary intestinal lymphangiectasia with PLE include constrictive pericarditis [4] long-standing congestive heart failure [4,7] surgical interventions, such as the Fontan or Glenn procedure [4], familial cardiomyopathy, interatrial septal defect, pulmonary stenosis, and tricuspid regurgitation [9].

Despite constrictive pericarditis being principally idiopathic, less common explanations include infectious etiologies, connective/autoimmune tissue diseases, and malignancy [3]. Other rare causes include CACP syndrome, Myhre syndrome, and IgG4-related disease [3]. Notably, secondary PLE caused by constrictive pericarditis can appear radiologically and histologically equivalent to primary intestinal lymphangiectasia, making differentiation challenging [9].

**Figure 2:**
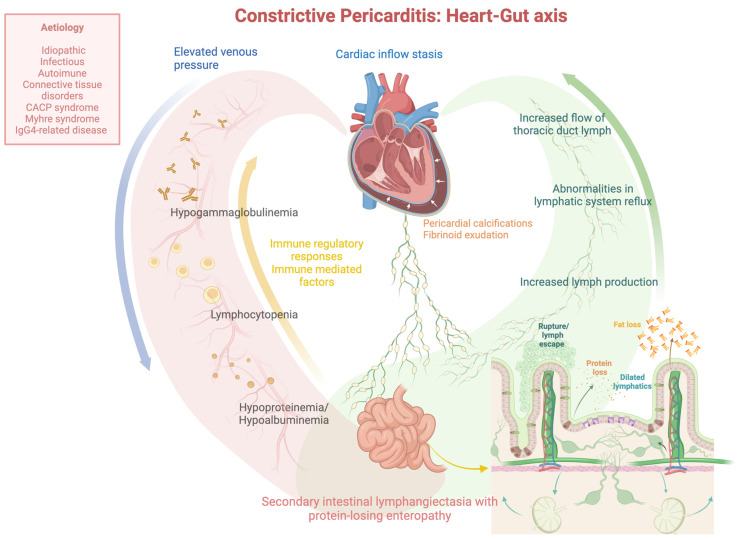
The pathophysiological mechanism connecting the heart–gut axis responsible for protein-losing enteropathy (PLE) generated by intestinal lymphangiectasia secondary to constrictive pericarditis. *CACP: camptodactyly-arthropathy-coxa vara-pericarditis syndrome*.

Figure 2 depicts the pathophysiological mechanism that links the heart and gut and is responsible for PLE generated by intestinal lymphangiectasia secondary to constrictive pericarditis.

In such cases, cardiac causative factors that may provoke intestinal protein loss include cardiac inflow stasis, increased lymph production with the subsequent formation of dilated lymphatics, and protein and fat loss due to rupture or lymph escape via transudation into the small bowel. The concept that constrictive pericarditis also causes a derangement in the production and flow of thoracic duct lymph is also supported by studies of experimental pericarditis in dogs [7]. However, elevated venous pressures are not the only cause, as demonstrated by Strober et al., who reported seven patients with equally severe right heart failure and systemic venous hypertension but no intestinal protein loss [24]. Additionally, Davidson et al. [25] discovered hypoalbuminemia and intestinal protein loss in a case of interatrial septa defect with high venous pressure. Similarly, Jeejeebhoy et al. uncovered the same in a case of pulmonary stenosis [26]. However, four patients with PLE had tricuspid regurgitation and rheumatic heart disease [24]. In addition, relative thoracic tract obstruction may become relevant in conditions of increased lymphatic flow [5]. For instance, radiation-induced constrictive pericarditis has been linked to the development of PLE following mediastinal irradiation for Hodgkin’s disease [27] or breast carcinoma [28].

Specifically, constrictive pericarditis causes symmetric lower extremity oedema, unlike the asymmetric distribution seen in intestinal lymphangiectasia [9]. In uncomplicated constrictive pericarditis without hypoproteinemia, ascites is more common than peripheral oedema [9]. 

Patients with intestinal lymphangiectasia and PLE may have a severe cellular immune deficiency that could raise infection hazards, presumably due to excessive lymphocyte and immunoglobulin depletion in the GI tract. Interestingly, all PLE with lymphocytopenia display lymphatic aberrations, while non-lymphocytopenic forms of GI protein loss do not [16]. Reversing protein loss inverts lymphocytopenia in PLE [16]. Additionally, lymphocytopenia occurs due to lymphocyte loss into the gut, causing anergy and masking an underlying tuberculous etiology of constrictive pericarditis [9].

An endoscopic biopsy can reveal dilated lacteals and lymphangiectasia and exclude other causes. In addition, the videocapsule endoscopy and double-balloon enteroscopy techniques are very useful for detecting and localizing precisely small bowel lymphangiectasias distally to the Treitz ligament, where a conventional endoscopy has no access, and for excluding other causes of PLE, though they are not available in all centers [4]. However, because lymphangiectasias may have a patchy distribution, normal endoscopic or histological findings do not rule out diagnoses. Additionally, radiologic GI findings in constrictive pericarditis are identical to those of primary intestinal lymphangiectasia, including mucosal oedema and thickening of the mucosal folds. These dilated submucosal and serosal lymphatic channels are more prominent in the duodenum and jejunum [9].

Echocardiography has a central role in the initial diagnosis of constrictive pericarditis. Peculiar signs, such as the abnormal inspiratory shift in the interventricular septum, increased respiratory variations in transmitral, trans tricuspid, and hepatic vein flow velocities, and the normality of early diastolic relaxation velocity (e’) at tissue Doppler imaging, increase the likelihood of the disease. These signs express increased ventricular interdependence and dissociation between intrathoracic and intracardiac pressures typical of pericardial constriction. For further diagnosis, CT and CMR are used to identify the presence of pericardial thickening. Invasive cardiac catheterization is indicated in dubious cases (in which the diagnosis is not reached by noninvasive diagnostic tools, i.e., echocardiography, CT scan, and/or CMR) and in assessing the severity of hemodynamic abnormalities, especially in cases with surgical indication. Cardiac catheterization exhibits a deep diastolic dip in the right ventricular pressure curve and a sharp rise in intraventricular pressure to a plateau when the rigid pericardium precludes further filling [1]. On the other hand, cardiac catheterization may miss the diagnosis in further infrequent cases, thus necessitating the support of CMR with/without a CT scan.

Regardless, the techniques and diagnostic criteria for constrictive pericarditis have not changed or evolved significantly over time, except for the replacement of previous radioisotopic techniques (131I-polyvinylpyrrolidone, 131I-albumin tests, and 51Cr albumin stool clearance) with Tc-GSA scintigraphy for PLE diagnosis.

A pericardiectomy is indicated in symptomatic patients meeting the diagnostic criteria of constrictive pericarditis and can improve the prognosis [29].

To assess GI protein loss, the fecal alpha1-antitrypsin is usually quantified in a random stool sample or, more accurately, by measuring alpha1-antitrypsin clearance in a 24-h stool collection with simultaneous serum measurement [4].

Moreover, excess radioisotopically labelled macromolecules such as 131I albumin, 131I polyvinylpyrrolidone, 51Cr albumin, and 67Cu ceruloplasmin in stool specimens have been frequently utilized in the recent decades to interpret PLE [9].

MRI or CT can be used to visualize the lymphangiectasias and reveal features distinctive of a specific causality.

However, a pericardiectomy to correct the cardiac disorder remains an effective procedure for constrictive pericarditis because it could potentially reverse the GI lymphatic dysfunction, thus improving the clinical syndrome associated with PLE and its outcomes, although its ability to invert extremely severe hypoalbuminemia with PLE is currently unclear.

## 6. Conclusions

Secondary intestinal lymphangiectasia associated with PLE occurs infrequently in constrictive pericarditis, as evidenced by the lack of clinical studies published in the analyzed literature.

Additionally, due to its significant clinical implications, special attention should be paid to determining the etiology of constrictive pericarditis by thoroughly examining the history of infection exposures, autoimmune diseases, previous cardiac surgery, congenital syndromes, and underlying cancer.

Ultimately, although there are infrequent cases that do not respond adequately to surgery, the cardio-intestinal abnormalities of PLE secondary to constrictive pericarditis are commonly reversed after a complete pericardiectomy.

## Figures and Tables

**Table 2:** Clinical characteristics of adult patients affected by protein-losing enteropathy (PLE) conditions secondary to constrictive pericarditis.

AUTHOR/YEAR	Petersen, V.P., 1963 (Ref. [7])	Wilkinson, P., 1965 (Ref. [22])	Müller, C., 1991 (Ref. [5])	Nikolaidis, N., 2005 (Ref. [1])	Meijers, B.K., 2006 (Ref. [23])	Chamouard, P., 2006 (Ref. [8])	Kikuchi, S., 2013 (Ref. [2])	Moriyama, H., 2016 (Ref. [6])
**AGE (years)/GENDER**	25/M	59/M	41-M	76-M	74-M	35-F	>70-M	38-M
**COMORBIDITIES**	cardiac cirrhosis	congestive heart failure	N	tuberculous nephritis history, atrial fibrillation, congestive heart failure, diabetes	past coronary bypass grafting, atrial fibrillation	pleuropericarditis history	N	mitral valve replacement history (severe mitral regurgitation/infective endocarditis)
**ONSET OF SYMPTOMS**	1 year	2 years	N	2 months	NA	19 years	NA	6 years
**ENDOSCOPY**	N	Y	Y	Y	Y	Y	Y	Y
**VIDEOCAPSULE**	N	N	N	N	N	mucosal edema, white curved lines associated with a combed aspect	N	N
**PLE LOCATION**	Jejunum	Jejunum	Jejunum	Duodenum	NA	Jejunum	NA	NA
**INTESTINAL HISTOLOGY**	autopsy: thickening/edema of peritoneum and small intestine; enlarged/swollen valves of Kerkring and villi containing foamy lipophages (expansion of the mucosal/submucosal lymphatic vessels)	dilatation of the lymphatic vessels of the villi	normal	markedly dilated lymphatics (intestinal lymphangiectasia)	N	NA	normal	NA
**LIVER HISTOLOGY**	mild portal cirrhosis (cardiac)	no cirrhosis	N	liver fibrosis	N	N	N	N
**X-RAY—CHEST**	pericardial calcifications	cardiomegaly	pleuropericardial adhesions	cardiomegaly, pleural effusions	N	N	pleural effusions	N
**X-RAY—BARIUM ENEMA**	proximal jejunal loops, slightly dilated; coarse mucosal folds	N	normal	N	N	non-specific coarsening of jejunum mucosal folds	N	N
**FECAL ALPHA1-ANTITRYPSIN CLEARANCE**	N	N	increased	N	N	increased	N	N
**RADIOISOTOPIC TECHNIQUES**	131I-polyvinylpyrrolidone, and 131I-albumin tests: reduced pool of exchangeable/high fractional turnover of serum-albumin	131I-albumin tests: reduced pool of exchangeable; 51Cr albumin stool clearance, increased	51Cr albumin stool clearance, increased	Tc-GSA scintigraphy, radionuclide accumulation in the intestine	Tc-GSA scintigraphy, radionuclide accumulation in the intestine	N	Tc-GSA scintigraphy, radionuclide accumulation in the intestine	Tc-GSA scintigraphy, radionuclide accumulation in the intestine
**ECG**	low voltage and inversion of T-waves	wandering atrial pacemaker, low-voltage QRS	NA	atrial fibrillation with occasional premature ventricular complexes	NA	NA	NA	NA
**ECOCARDIOGRAPHY**	N	N	thickened pericardium	N	thickened pericardium	inferior vena cava ectasia	thickened pericardium	thickened pericardium with calcifications, biatrial enlargement, mild mitral valve regurgitation
**CT SCAN**	N	N	N	thickened/calcified pericardium, hepatomegaly	N	slight ascites	thickened pericardium	bilateral pleural effusion and thickened pericardium
**MRI**	N	N	thickened pericardium, tubular-shaped right ventricle	thickened and calcified pericardium, hepatomegaly	N	thickened pericardium	N	N
**RIGHT HEART CATHETERIZATION**	Y	Y	Y (missed diagnosis)	Y	Y	Y	Y	Y
**PERICARDIECTOMY**	Y	Y	Y (subtotal)	Y	Y	Y (subtotal)	Y	Y
**CONSTRICTIVE PERICARDITIS-HISTOLOGY**	Y	no typical features	Y	Y	Y	Y	Y	Y
**ETIOLOGY**	unknown	unknown	unknown	unknown	unknown	unknown	unknown	unknown
**RESPONSE TO PERICARDIECTOMY**	edema and ascites were absent for six years, a slight cardiac inflow-stasis persisted and developed a permanent hypoalbuminemia	congestive heart failure and hypoalbuminemia improved, small bowel histology normalized, but liver function did not completely improve	peripheral edema disappeared and serum protein normalized, despite the persistence of a small enteric protein loss one year after surgery	anemia, hypoproteinemia, hypertriglyceridemia ameliorated	cardiac conditionimproved, PLE resolved, serum albumin increased	serum albumin normalized in two months	leg oedema/pleural effusion disappeared, serum albumin normalized	leg oedema and pleural effusion resolved, albumin. levels normalized in three months

N: No; Y: yes; NA: not available; PLE: protein-losing enteropathy; Tc-GSA: technetium-99m diethylenetriamine pentaacetic acid galactosyl human serum albumin; ECG: electrocardiogram; MRI: magnetic resonance imaging.
